# Lifetime progression of IgA nephropathy: a retrospective cohort study with extended long-term follow-up

**DOI:** 10.1186/s12882-025-03958-y

**Published:** 2025-01-21

**Authors:** Mariell Rivedal, Ole Petter Nordbø, Yngvar Lunde Haaskjold, Rune Bjørneklett, Thomas Knoop, Øystein Eikrem

**Affiliations:** 1 Department of Clinical Medicine, University of Bergen, Bergen, Norway; 2 Department of Medicine, Haukeland University Hospital, Bergen, Norway; 3 Emergency Care Clinic, Haukeland University Hospital, Bergen, Norway

**Keywords:** Chronic renal failure, ESKD, IgA nephropathy, Glomerulonephritis, Proteinuria

## Abstract

**Background:**

IgA nephropathy (IgAN) exhibits an unpredictable trajectory, creating difficulties in prognostication, monitoring, treatment, and research planning. This study provides a comprehensive depiction of the progression of kidney function throughout the disease course, from diagnosis to a span of 36 years post-diagnosis.

**Methods:**

We utilized a cohort of 400 Norwegian IgAN patients, from diagnosis to the occurrence of death, initiation of kidney replacement therapy (KRT), or the latest follow-up. Recorded proteinuria (*n* = 2676) and creatinine (*n* = 8738) measurements were retrieved. Patients were divided into subgroups based on their specific estimated glomerular filtration rate (eGFR) slopes.

**Results:**

Median follow-up was 16 years. During this period, 34% of patients either died or initiated KRT. Among patients who reached endpoint, the median duration from diagnosis to the initiation of KRT or death was 8 years. Notably, 34% of the cohort exhibited a stable disease course, characterized by an eGFR decline of less than 20% between two consecutive measurements. Differences in subsequent disease trajectories among two subgroups with similar eGFR levels at diagnosis could not be accounted for by variations in treatment strategies. Among patients with proteinuria < 1 g/24 h in less than half of the measurements, KRT was five times more prevalent compared to those with more than half of the measurements recording proteinuria < 1 g/24 h (*p*-value = 0.001).

**Conclusions:**

While a significant proportion of IgAN patients reach kidney failure within their lifetimes, outcomes vary widely. Clinical data at diagnosis offer limited insights into long-term risks. Enhanced risk stratification necessitates data collection at multiple time points.

**Supplementary Information:**

The online version contains supplementary material available at 10.1186/s12882-025-03958-y.

## Introduction

Immunoglobulin A nephropathy (IgAN) is the most prevalent primary glomerulonephritis worldwide and a significant cause of kidney failure [1]. It predominantly affects relatively young individuals [2–4]. It is crucial for the patient and their physician to understand the long-term prognosis, but prognostication is complicated by the heterogeneous nature of the disease [1, 3, 5]. Accurate prognosis is essential for personalizing follow-up and therapy, which is especially important now that there is an increasing number of ongoing clinical trials for promising novel therapies [3].

The Kidney Disease: Improving Global Outcomes (KDIGO) guidelines for glomerular diseases [6] recommend the use of the International IgAN Prediction Tool (IIgANPT) [7–10] for risk stratification. While this tool is valuable for predicting the risk of end-stage kidney disease (ESKD) or a > 50% decline in estimated glomerular filtration rate (eGFR), it can only predict the risk of kidney failure up to 6.7 years after diagnosis. This duration is relatively short compared to the remaining lifespan of the typical patient with IgAN [3]. Some subgroups have a benign disease presentation, with a low estimated risk of progression, yet develop progressive kidney failure [5]. Consequently, certain patients undergo suboptimal treatment and follow-up because of the presumed benign nature of their condition, while others undergo unnecessary treatments based on the presumption of a progressive disease course.

To improve the therapeutic approaches of patients with IgAN, a deeper understanding of the disease course is necessary. Although several studies on long-term outcomes exist [3, 5, 11–15], none provide detailed information on the development of eGFR and proteinuria throughout the entire disease course.

Hence, this study set out to evaluate the distinctive features and progression of IgAN among Norwegian patients over an extended follow-up period. By meticulously tracking the evolution of eGFR and proteinuria, we aim to provide a comprehensive analysis that reveals the different long-term disease trajectories of IgAN, thereby enhancing our understanding of its heterogeneous disease course.

## Materials and methods

This retrospective, multicenter, observational cohort study adheres to the guiding principles of the Declaration of Helsinki and received approval from the Regional Ethics Committee of Western Norway (No. 2013/553). Informed consent was obtained from all participants.

### Study population

We identified 462 adults with biopsy-verified IgAN, diagnosed in Western Norway between 1988 and 2015. We excluded 49 patients with missing follow-up data, due to short follow-up (required KRT or died within 6 months after diagnosis) or because the patient moved away from the Western Norwegian region shortly after diagnosis. We excluded 6 patients due to inability to contact them for consent and 7 patients who declined participation. Ultimately, 400 patients were included.

Baseline data were obtained from the Norwegian Kidney Biopsy Registry. Follow-up data were retrieved from patient records. The follow-up period extended from diagnosis until either the initiation of KRT, death, or the end of follow-up in February 2024.

We obtained serum creatinine measurements throughout the follow-up period. The eGFR was calculated using the Chronic Kidney Disease Epidemiology Collaboration equation (CKD-EPI) [16] for adults, and the Chronic Kidney Disease (CKD) in Children under 25 calculator [17, 18] for pediatric patients. Based on the eGFR values, we divided the patients into subgroups. Patients with an unstable disease course had ≥ 20% eGFR decline between two subsequent measurements, with < 10 years between the two measurements; otherwise, the disease course was deemed stable. This definition is based on the KDIGO Clinical Practice Guideline for the Evaluation and Management of CKD [19], which recommends that an absolute change in eGFR ≥ 20% in a patient with CKD should be further evaluated, as it surpasses expected variability.

We also retrieved proteinuria values throughout the follow-up period until eGFR was < 15 mL/min/1.73 m^2^. All values are presented as g/24 h. A urinary protein-creatinine ratio of 100 mg/mmol was considered comparable with protein excretion of 1 g/24 h [20].

### Statistical analyses

Data were processed using the R software (version 4.2.1). Statistical significance was defined as *p*-value < 0.05.

Categorical variables are shown as frequencies and percentages, while continuous variables are represented by medians (with interquartile ranges [IQR]). Statistical differences were assessed using the Mann–Whitney U test for continuous data and Fisher’s exact test for categorical data, assuming independence between the compared variables and without adjusting for confounders. We employed multivariate survival analysis using Cox regression to investigate factors associated with the risk of ESKD or death.

Patient-specific longitudinal trajectory plots were used to represent the eGFR changes during the follow-up period. Based on the changes between two consecutive measurements, we divided the patients into subgroups (stable or unstable disease course). An eGFR of ≥ 90 mL/min/1.73 m^2^ at diagnosis was referred to as normal kidney function, while an eGFR within the range of 60–89 mL/min/1.73 m^2^ was classified as mildly decreased, and an eGFR < 60 mL/min/1.73 m^2^ at diagnosis was defined as moderately/severely decreased. Kaplan–Meier analyses were conducted to compare kidney survival times between different patient subgroups. Log-rank tests were used to determine statistical significance.

## Results

### Patient characteristics

A total of 400 patients were included (Supplementary Figure S1). The indications for a kidney biopsy throughout the retrospective study period of 1988–2015 followed the current standard of care throughout this period. The clinicians could report several indications for each case, and the two most common were hematuria (*n* = 316) and proteinuria (*n* = 266).

Most patients (72%) were male, typical for Norwegian IgAN cohorts [5, 21]. At diagnosis, median age was 36 years, median eGFR was 75 mL/min/1.73 m^2^, and median proteinuria was 1.0 g/24 h. A third of the patients (36%) had hypertension, defined as systolic blood pressure > 140 mm Hg and/or diastolic blood pressure > 90 mm Hg. However, only 74 patients (18%) used Renin–Angiotensin–Aldosterone system (RAAS) inhibitors at diagnosis.

Further details are presented in Table 1.

**Table 1:** Baseline characteristics

Variable	All patients (*n* = 400)
***Age at diagnosis, n (%)***	*400 (100)*
Median, years (IQR)	36 (25–50)
***Sex, n (%)***	*400 (100)*
Female	113 (28)
Male	287 (72)
***Systolic blood pressure at diagnosis, n (%)***	*381 (95)*
Median, mm Hg (IQR)	132 (120–148)
***Diastolic blood pressure at diagnosis, n (%)***	*380 (95)*
Median, mm Hg (IQR)	80 (75–90)
***Middle arterial pressure at diagnosis, n (%)***	*380 (95)*
Median, mm Hg (IQR)	98 (93–110)
***Hypertension at diagnosis, n (%)***	*380 (95)*
Yes	138 (36)
No	242 (64)
***Body mass index at diagnosis, n (%)***	*278 (70)*
Median, kg/m^2^ (IQR)	24.3 (22.2–27.2)
***Indication for kidney biopsy, n (%) – Can be more than one per patient***	*400 (100)*
Nephrotic syndrome	27 (7)
Nephritic syndrome	28 (7)
Suspicion of rapidly progressive glomerulonephritis	2 (0.5)
Proteinuria	266 (67)
Hematuria	316 (79)
***Proteinuria at diagnosis, n (%)***	*367 (92)*
Median, g/24 h (IQR)	1.0 (0.4–2.0)
***Hematuria at diagnosis – urinary dipstick***^a^***, n (%)***	*335 (84)*
0 (< 10 cells/μL)	19 (6)
1 (10–24 cells/μL)	150 (45)
2 (25–49 cells/μL)	33 (10)
3 (50–249 cells/μL)	91 (27)
4 (≤ 250 cells/μL)	42 (13)
***eGFR at diagnosis, n (%)***	*396 (99)*
Median, mL/min/1.73 m^2^ (IQR)	75 (52–98)
***CKD stage at diagnosis, n (%)***	*396 (99)*
Stage 1	134 (34)
Stage 2	132 (33)
Stage 3a	53 (13)
Stage 3b	42 (11)
Stage 4	27 (7)
Stage 5	8 (2)
***RAAS inhibitor at diagnosis, n (%)***	*400 (100)*
Yes	74 (18)
No	326 (82)
***Corticosteroids at diagnosis, n (%)***	*400 (100)*
Yes	2 (0.5)
No	398 (99.5)
***Glomeruli in diagnostic kidney biopsy, n (%)***	*400 (100)*
Median, n (IQR)	12 (8–16)
***Proportion of sclerotic glomeruli in diagnostic kidney biopsy, n (%)***	*262 (66)*
Median, % (IQR)	14 (0–30)

For quantitative variables, values are expressed as medians (interquartile ranges [IQR]), and for qualitative variables, values are expressed as n (%). *eGFR* Estimated glomerular filtration rate. *CKD* Chronic kidney disease. ^a^The standard urinary dipstick is the Combur-Test® strip (Roche Diagnostics)

### Outcomes

Median follow-up was 16 years. During this period, 69% received RAAS inhibitors (Supplementary Figure S2), 20% received corticosteroids, and 7% received sodium-glucose cotransporter 2 (SGLT2) inhibitors.

**Fig. 1:**
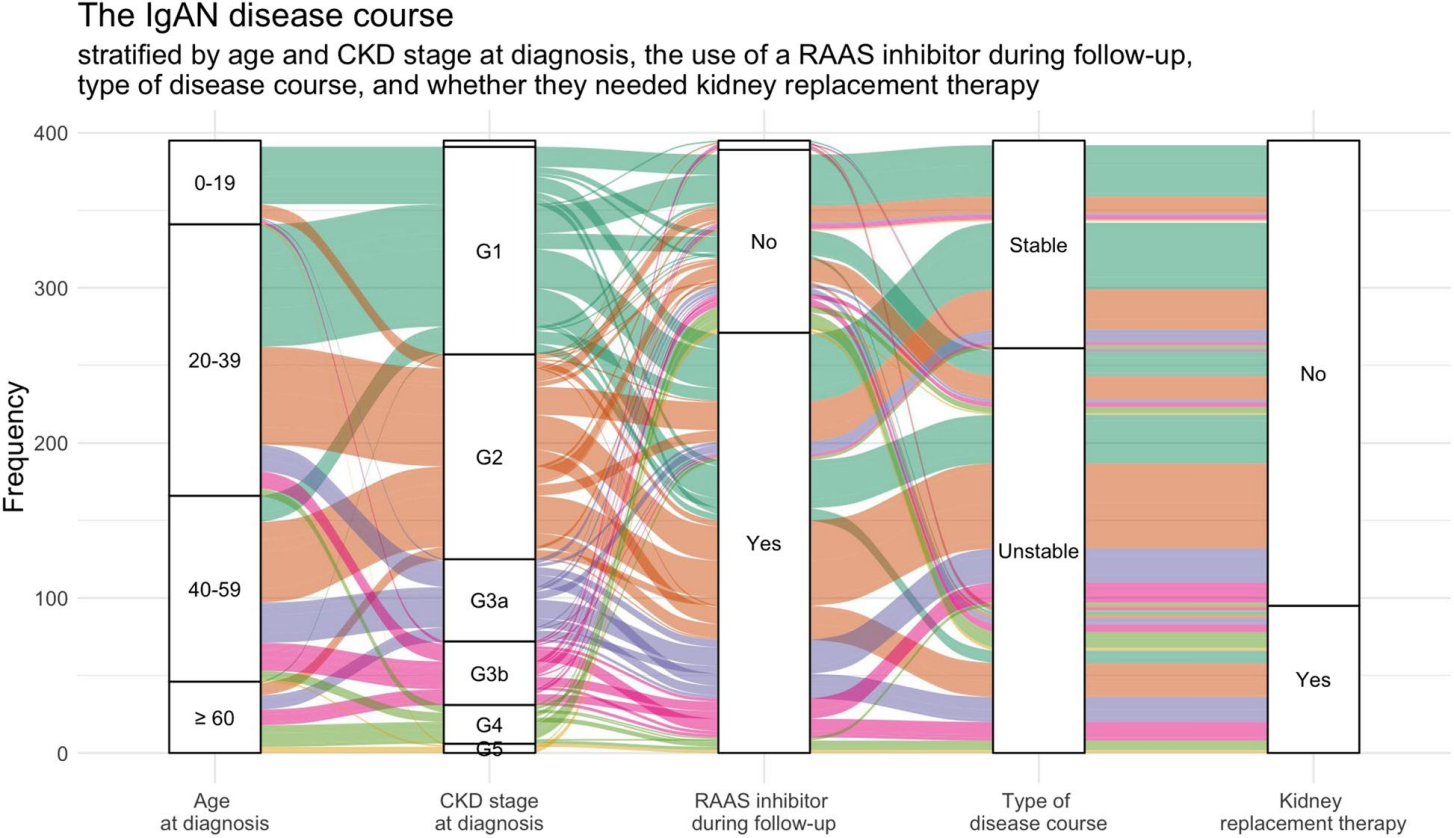
The IgAN disease course*.* Alluvial diagram describing the disease course for the patients in this cohort, stratified by age and CKD stage at diagnosis, type of disease course and whether they needed kidney replacement therapy. The colors represent CKD stage at diagnosis (G1 = mint, G2 = orange, G3a = purple, G3b = pink, G4 = green, G5 = yellow, unknown = grey). CKD = Chronic kidney disease. RAAS = Renin–angiotensin–aldosterone system

This cohort showed a great variation in disease courses (Fig. 1). Many patients experienced a significant decline in kidney function, with 34% reaching ESKD and/or dying during the follow-up period. KRT was initiated in 25%. Among the patients who reached an endpoint, the median time from diagnosis until KRT or death was 8 years, and those patients’ median age at endpoint was 57 years. The survival rates (patients who had not reached death or KRT) at 10- and 20-years post-biopsy were 79% and 67%, respectively.

Further details are presented in Table 2.

**Table 2:** Clinical outcomes during follow-up

Variable	All patients (*n* = 400)
***Length of follow-up, n (%)***	*400 (100)*
Median, years (IQR)	16 (9–24)
***Use of RAAS inhibitor during follow-up, n (%)***	*392 (98)*
Yes	272 (69)
No	120 (31)
***Use of corticosteroids during follow-up, n (%)***	*394 (99)*
Yes	78 (20)
No	313 (78)
***Use of SGLT2 inhibitor during follow-up, n (%)***	*396 (99)*
Yes	28 (7)
No	368 (92)
***Time-averaged proteinuria (g/24 h), n (%)***	*341 (85)*
Median (IQR)	1
***Proportion of follow-up period with proteinuria* < *1 g/24 h, n (%)***	*341 (85)*
Median, % (IQR)	67 (22–90)
***Proportion of measurements with proteinuria* < *1 g/24 h, n (%)***	*341 (85)*
Median, % (IQR)	71 (43–92)
***ESKD or death event, n (%)***	*400 (100)*
Yes	135 (34)
No	265 (66)
***Death event, n (%)***	*400 (100)*
Yes	69 (17)
No	331 (83)
***Death event without KRT, n (%)***	*69 (17)*
Yes	34 (49)
No	35 (51)
***ESKD, n (%)***	*400 (100)*
Yes	101 (25)
No	299 (75)
***KRT, n (%)***	*400 (100)*
Yes	99 (25)
No	301 (75)
***First event, n (%)***	*133 (33)*
Death	34 (25.6)
Dialysis	78 (58.6)
Transplant	21 (15.8)
***Years until first event, n (%)***	*135 (34)*
Median, years (IQR)	8 (5–14)
***Age at first event, n (%)***	*135 (34)*
Median, years (IQR)	57 (40–68)
***Survival rate (have not reached death or KRT), estimate (95% CI), n (%)***	*400 (100)*
5-year	91 (88–93)
10-year	79 (76–84)
15-year	73 (69–78)
20-year	67 (62–72)
25-year	62 (56–68)
30-year	55 (49–63)
***Quartile survival estimate, years (95% CI), n (%)***	*400 (100)*
90%	6 (4–7)
80%	10 (8–14)
70%	18 (15–23)
60%	27 (23-NE)
50%	NE (29-NE)

For quantitative variables, values are expressed as medians (interquartile ranges [IQR]), and for qualitative variables, values are expressed as n (%). RAAS = Renin–angiotensin–aldosterone system. SGLT2 = Sodium glucose cotransporter 2. KRT = Kidney replacement therapy. *ESKD* End-stage kidney disease. *CI* Confidence interval. *NE* Not estimable

We employed multivariate survival analysis using Cox regression to investigate factors associated with the risk of ESKD or death. A total of 288 patients were included, as missing data were handled through exclusion. Unsurprisingly, age, CKD stage and proteinuria at diagnosis were the only variables that significantly affected the risk of ESKD or death (Table 3).

**Table 3:** Multivariate Survival Analysis on factors affecting the risk of ESKD or death

Variable	HR	95% CI	*p*-value
Gender (male)	1.23	0.77–1.97	0.395
Age at diagnosis (years)	1.02	1.00–1.03	0.043
CKD stage at diagnosis (G2 compared to G1)	2.40	1.10–5.23	0.028
CKD stage at diagnosis (G3a compared to G1)	4.64	1.87–11.50	< 0.001
CKD stage at diagnosis (G3b compared to G1)	8.49	3.56–20.25	< 0.001
CKD stage at diagnosis (G4 compared to G1)	13.72	5.57–33.79	< 0.001
CKD stage at diagnosis (G5 compared to G1)	27.90	6.42–121.22	< 0.001
Proteinuria at diagnosis (g/24 h)	1.17	1.08–1.27	< 0.001
Hematuria at diagnosis (yes)	1.05	0.38–2.93	0.920
Systolic blood pressure at diagnosis (mm Hg)	1.01	0.99–1.02	0.279
Diastolic blood pressure at diagnosis (mm Hg)	0.98	0.95–1.00	0.084
Use of RAAS inhibitor during follow-up (yes)	0.69	0.43–1.12	0.138
Use of immunosuppression during follow-up (yes)	1.28	0.77–2.12	0.335

*ESKD* End-stage kidney disease. *CKD* Chronic kidney disease. *RAAS* Renin–angiotensin–aldosterone system. *HR* Hazard ratio. *CI* Confidence interval

### Kidney function throughout the IgAN disease course

We retrieved 8738 eGFR values during the follow-up period. After excluding patients with < 10 creatinine measurements, 395 patients were included in the subsequent analyses.

**Fig. 2:**
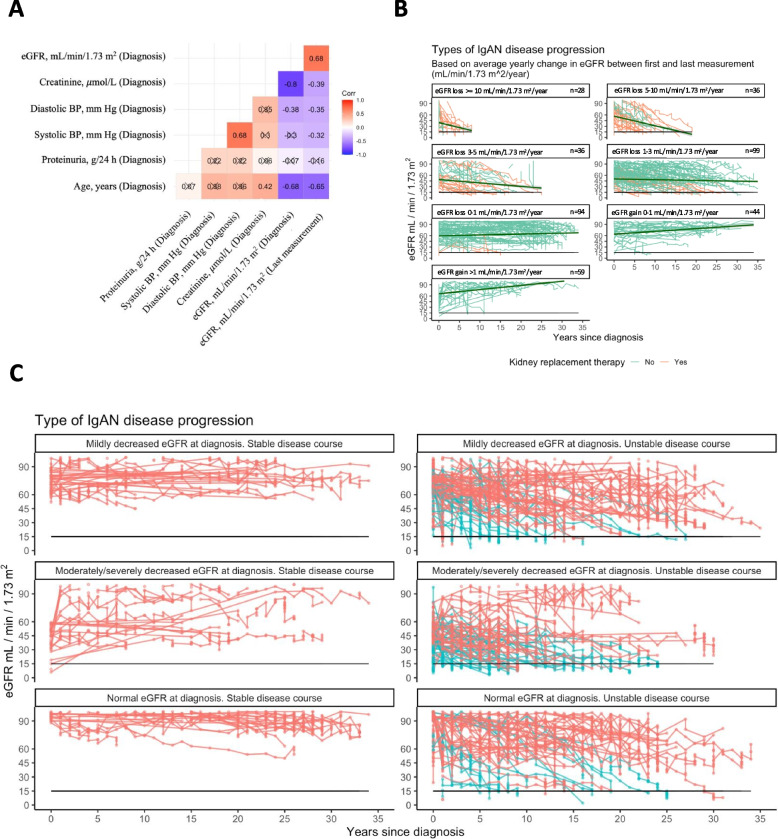
*eGFR throughout the IgAN disease course.* **A** Correlation matrix. Crossed out values reveal correlations that are not statistically significant. **B** Visual representation of the kidney function throughout the IgAN disease course based on average yearly change in eGFR between first and last measurement. We have divided the patient’s disease courses in seven subgroups based on the average eGFR slopes. The eGFR in each measurement is represented on the x-axis, and the y-axis represents years after diagnostic kidney biopsy. The dark green lines represent a linear model with 95% confidence interval per subgroup. Patients with a light green eGFR slope did not need kidney replacement therapy at the end of follow-up, while those with an orange eGFR slope initiated kidney replacement therapy after the last measurement. **C** Visual representation of the kidney function throughout the IgAN disease course. The eGFR in each measurement is represented on the x-axis, and the y-axis represents years after diagnostic kidney biopsy. The two main groups of the cohort, patients with a stable (*n* = 134) or an unstable (*n* = 261) disease course, have been further divided into several subgroups, based on the patient´s eGFR at the time of diagnosis (Normal: ≥ 90 mL/min/1.73 m^2^; Mildly decreased: 60–89 mL/min/1.73 m^2^; Moderately/severely decreased: < 60 mL/min/1.73 m^2^). Red lines indicate the eGFR slopes of patients who did not need kidney replacement therapy at the end of the follow-up period. Blue lines indicate the eGFR slopes of those who initiated kidney replacement therapy during follow-up, with the last eGFR measurement being the last one before initiation of dialysis or kidney transplantation. The black line in each graph represents eGFR = 15 mL/min/1.73 m^2^. CKD = Chronic kidney disease. eGFR = Estimated glomerular filtration rate. BP = Blood pressure

There was a moderate, positive relationship between eGFR at diagnosis and at the last measurement (*r* = 0.68, *p*-value < 0.05) and a moderate, negative relationship between age at diagnosis and the last eGFR measurement (*r* = -0.65, *p*-value < 0.05) (Fig. 2A). There was also a low, negative relationship between diastolic (*r* = -0.35, *p*-value < 0.05) or systolic (*r* = -0.32, *p*-value < 0.05) blood pressure at diagnosis and the last eGFR measurement.

The cohort demonstrated substantial variation in eGFR slopes (Fig. 2B). Only 7% experienced a decline of > 10 mL/min/1.73 m^2^/year. The most common rate of eGFR loss was an average of 1–3 mL/min/1.73 m^2^/year (25%). Interestingly, 26% experienced improvement of their kidney function, when comparing the first and last measurements. Among the patients who needed KRT after more than 1 year (*n* = 92), the median average eGFR slope was -3.2 mL/min/1.73m^2^/year (IQR -5.4-(-1.6)). Patients who did not need KRT, and who had more than 1 year of follow-up (*n* = 140), had an average eGFR slope of -0.8 mL/min/1.73m^2^/year (IQR -1.5-(-0.1)).

We divided the cohort into subgroups based on eGFR at diagnosis and whether their subsequent disease course was stable (34%) or unstable (66%) (Fig. 2C). Among the patients with an eGFR of ≥ 90 mL/min/1.73 m^2^ at diagnosis, 57% experienced an unstable disease course. In cases where the eGFR at diagnosis fell within the range of 60–89 mL/min/1.73 m^2^, 71% encountered subsequent unstable disease courses. Notably, a minority of 36 patients were diagnosed with moderately/severely decreased kidney function, yet remarkably sustained a stable disease course. However, some of these patients presented with a significantly decreased kidney function, which is later improved to a stable level of eGFR > 60 mL/min/1.73 m^2^. It is unsurprising that a diagnosis at this late stage significantly increased the likelihood of experiencing an unstable disease course, evidenced by most cases (n = 91) following such a pattern.

### Proteinuria throughout the disease course

We retrieved 2676 proteinuria measurements during the follow-up period. Patients with ≥ 2 proteinuria measurements (*n* = 365) were included in the subsequent analyses.

Median proteinuria during follow-up was 0.6 g/24 h. During follow-up, each patient had proteinuria < 1 g/24 h in a median of 71% of the measurements (Table 2). Interestingly, the differences between patients with a stable and an unstable disease course were not statistically significant (*p*-value = 0.8).

**Fig. 3:**
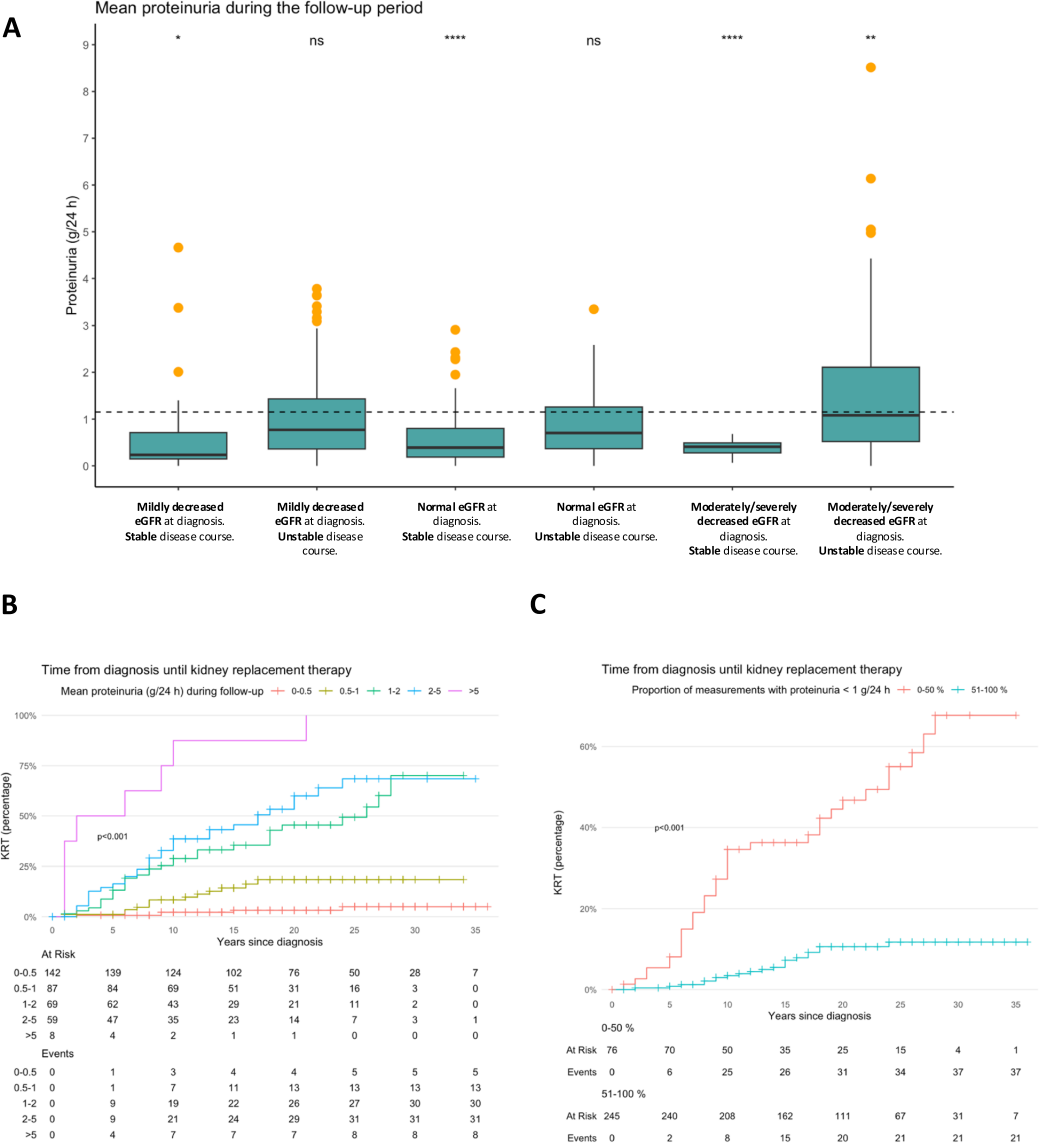
*Proteinuria throughout the IgAN disease course.* **A** Mean proteinuria (g/24) during follow-up. Orange points equal outliers. Dashed line symbolizes base mean (1.2 g/24 h). ns = Not significant. * = *p*-value (0.01, 0.05]. ** = *p*-value (0.001, 0.01]. **** = *p*-value < 0.001. **B** Kaplan–Meier plot describing the differences in time from diagnosis until initiation of kidney replacement therapy based on mean proteinuria (g/24 h) during follow-up. Censored cases are marked with a cross. **C** Kaplan–Meier plot describing the differences in time from diagnosis until initiation of kidney replacement therapy based on the proportion measurements with proteinuria < 1 g/24 h. Censored cases are marked with a cross. KRT = Kidney replacement therapy

All subgroups with a stable disease course had significantly lower mean proteinuria compared to the entire cohort (Fig. 3A). The subgroup with the lowest proteinuria during follow-up comprised patients who had moderately/severely decreased eGFR at diagnosis (*n* = 36), with a subsequent stable disease course, with a mean proteinuria of 0.4 g/24 h. Patients with the highest proteinuria during follow-up belonged to the subgroup with mildly decreased eGFR at diagnosis and a subsequent unstable disease course (*n* = 38), with a mean proteinuria of 1.6 g/24 h.

A Kaplan–Meier survival analysis was conducted to compare kidney survival time among subgroups based on mean proteinuria during follow-up (Fig. 3B). The survival distributions for the subgroups were significantly different (χ^2^(4) = 156, *p*-value < 0.001), with lower kidney survival in patients with high proteinuria. Pairwise log rank comparisons were conducted to determine which subgroups had different kidney survival distributions. After Bonferroni correction, there was a statistically significant difference in kidney survival distributions between all subgroups, except for between patients with a mean proteinuria of 1–2 g/24 h during follow-up and those with a mean proteinuria of 2–5 g/24 h (*p*-value = 0.22).

We also performed a Kaplan Meier analysis to compare kidney survival time based on whether a patient had proteinuria < 1 g/24 h in more or less than 50% of the measurements during the follow-up period. Log rank testing revealed that patients with proteinuria < 1 g/24 h in ≤ 50% of the measurements had worse kidney survival (*p*-value < 0.001) (Fig. 3C). KRT was five times as common among patients with proteinuria < 1 g/24 h in ≤ 50% of the measurements (49%), compared to > 50% of the measurements (9%) (*p*-value = 0.001). In patients with proteinuria < 1 g/24 h in ≥ 90% of the measurements (*n* = 145), 5 patients (3%) needed KRT.

## Discussion

In this extensive, multicenter cohort of Norwegian patients with IgAN, we have described the evolution of kidney function throughout the disease course. We hypothesized that IgAN has several different clinical profiles during long-term follow-up. This is the first study offering such a detailed depiction of eGFR and proteinuria trajectories spanning several decades post-diagnosis. We found that proteinuria < 1 g/24 in < 50% of the follow-up measurements was linked to poorer kidney survival, and patients with an unstable disease course exhibited higher proteinuria during follow-up. Age, CKD stage and proteinuria at diagnosis were the only variables that significantly affected the risk of ESKD or death, according to a multivariate regression analysis.

Until recently, therapeutic options for patients with IgAN have been limited [6]. With the anticipated approval of novel therapies [22–29], the need for personalized treatment approaches is becoming more pressing [30], underscoring the importance of conducting a proper risk assessment for each patient to select a personalized, cost-effective therapy.

eGFR decline correlates with disease progression [31], and the eGFR slope may serve as a surrogate endpoint in clinical trials and a predictor of treatment effect [32–37]. However, eGFR slopes often derive from randomized clinical trials with selected cohorts, excluding individuals with comorbid conditions like diabetes or cardiovascular disease [32] (Supplementary Table S1). Thus, for the eGFR slope to function as an adequate surrogate endpoint in clinical trials, and in clinical practice, we need increased information about the slopes in the natural disease course.

We have presented the significant variation in long-term eGFR slopes within a cohort of 400 patients with IgAN. Comparing patients with similar eGFR levels at diagnosis revealed diverse long-term disease courses. While some patients maintained stable kidney function over several decades, others experienced unstable disease courses, with or without progression to ESKD. This finding is of great importance regarding patient inclusion in future clinical trials, as the natural variability in disease progression could substantially influence study outcomes.

Therefore, a single time-point assessment of clinical characteristics, such as at diagnosis, is insufficient for predicting future disease progression. We have previously shown that some patients with assumed benign IgAN at diagnosis can experience a subsequent progressive disease course [5, 38]. In this cohort, the opposite is also true. Some patients present with moderately/severely decreased eGFR but keep a stable kidney function for the next three decades. Others present with acute kidney injury but regain their kidney function after some time, although current prediction tools would suggest that these patients would have poor prognoses.

Our data highlight the necessity of including longitudinal data beyond the initial diagnosis when assessing the risk of kidney failure. We are not the first to point out the weakness of only using diagnostic data in prognostication, as Barbour et al. have updated the IIgANPT so that it also can be used at one or two years post-biopsy and thus be used to evaluate a patient´s risk of disease progression after a period of observation with supportive care [10]. KDIGO guidelines also recommend further research to assess whether prediction tools, such as the IIgANPT, can be used to predict prognosis during the disease course and to evaluate response to therapy [6].

The only KDIGO-approved markers for disease progression are eGFR and proteinuria [6], and the IIgANPT can only predict risk up to a 6.7-year horizon [7, 8, 30]. These factors limit prognostication in IgAN. To improve prognostication, an option is to use longitudinal data, as our findings suggest. Chen et al. [39] recently developed a multidimensional deep learning model that accurately and dynamically predicts kidney outcomes in Chinese patients, indicating that longitudinal models may indeed be used to predict kidney outcomes in the future. A potential limitation with using follow-up data in prognostication is that it may delay risk stratification [40]. In a future clinical setting, it may therefore be necessary to include both models that use cross-sectional data, such as the IIgANPT, and models that use longitudinal data, for more accurate risk stratification.

Proteinuria, too, has emerged as a significant surrogate endpoint in IgAN drug trials [33, 35]. When considering proteinuria as a surrogate endpoint, both the extent of reduction and the duration of remission should be considered [41]. According to the KDIGO guidelines, the treatment target should be reduction of proteinuria to < 1 g/24 h [6]. However, a deeper understanding of long-term outcomes for patients maintaining proteinuria levels below this threshold is necessary [3].

Our findings indicate that mean proteinuria during follow-up was higher in patients with an unstable disease course. Although proteinuria tends to decrease when a patient reaches ESKD, due to the reduced glomerular filtration, this did not affect our data, since we stopped data acquisition when the patient reached ESKD.

Proteinuria < 1 g/24 in < 50% of the measurements was associated with poorer kidney survival compared to those with more consistent reductions (*p*-value < 0.001). Surprisingly, even among patients with proteinuria below the recommended 1 g/24 h in > 90% of the measurements, 5 patients needed KRT. While this suggests that the overall kidney survival is high among these patients, it also shows that sustained proteinuria reduction does not eliminate all risk of progression. This is supported by Tang et al. [42], who found that patients with IgAN and proteinuria ≥ 0.5 g/24 h also have an increased risk of kidney failure, especially those who had proteinuria ≥ 1 g/24 h before initiating therapy. Maybe there is no safe proteinuria, but the lower, the better, and any reduction of proteinuria is associated with better kidney outcomes [43].

Several studies on long-term outcomes exist [3, 5, 11–15]. However, the cohorts consist of patients from a single country, and geographic differences may affect outcomes [2, 44, 45]. In Supplementary Table S2, we have compared some of our findings with those in British (3), Chinese (11) and Japanese (14) studies on long-term outcomes. Importantly, our follow-up time is longer than for the three studies. Asian patients with IgAN generally have an increased risk of kidney failure [45, 46]. Interestingly, the 10-year survival rate in our cohort was comparable to those in the Chinese (11) and Japanese (14) cohorts, while the British cohort (3) had a much lower survival rate. This may be because the patients in the British cohort were older and had poorer kidney function at baseline. The British cohort only consisted of patients with eGFR < 60 mL/min/1.73 m^2^ and proteinuria > 0.5 g/24 h at enrolment, excluding those with a relatively good kidney function and those with isolated non-visible hematuria. Thus, the differences in clinical characteristics at diagnosis challenges comparison of long-term outcomes between different ethnicities and nations.

When studying long-term outcomes in a potentially slowly progressive disease, such as IgAN, it is important to be aware of the impact of treatment. During follow-up, only 69% received RAAS inhibitors, and 20% received corticosteroids. The low proportion of corticosteroids and RAAS inhibitors in this cohort has also been observed in previous studies, such as a Norwegian cohort with long follow-up [38], and in the discovery and validation cohorts that were used to develop the IIgANPT [7]. In this study, it may be due to the inclusion of patients with a wide range of disease courses, with only 7% experiencing an average eGFR loss of > 10 mL/min/1.73 m^2^/year. Regarding corticosteroid therapy, Norwegian physicians tend to be restrictive when considering this treatment [47], and it would not be indicated in patients with high eGFR and a stable disease. The reason for the low proportion of RAAS inhibitor use may be that many patients were not regularly followed by a nephrologist after diagnosis due to an assumed mild subsequent disease course, and they were not considered for therapy.

It is possible that treatment methods changed over time, due to the long enrollment period (1988–2015) in which treatment trends may have varied. However, we included a cohort with little treatment beyond supportive care, thus limiting this confounder. Moreover, we found that the differences in disease courses between subgroups with similar eGFR at diagnosis could not be explained by a lower presence of treatment among those with an unstable disease course (Supplementary Tables S3-S5).

Our study has some limitations. Since the study is retrospective, we lack detailed information about medication and lifestyle-related factors during follow-up. We also lack information about the existence of confounding variables that may affect eGFR and proteinuria at various time-points. The retrospective nature of the study also affected the quality of the kidney function parameters. To increase the number of proteinuria measurements, we considered a urinary protein-creatinine ratio of 100 mg/mmol to be comparable with protein excretion of 1 g/24, but this conversion may not be entirely accurate. Moreover, the CKD-EPI equation can only be applied with isotope dilution mass spectrometry traceable creatinine values, but this method was not yet established when the first creatinine measurements in our study was performed, thus introducing a possible source of error in the eGFR values [48]. Although the dataset included 2676 proteinuria measurements from 400 patients over a median follow-up period of 16 years, this averages to only 2–3 measurements per patient, thereby limiting the analytical possibilities. In this cohort, RAAS inhibitors were only used by 69% of the patients. This is partly because some for some patients, the follow-up period took place before RAAS inhibition was recommended by the KDIGO guidelines [6, 49]. This, and the fact that only 7% of the patients were treated with SGLT2 inhibitors, is not reflective of current real world clinical practice. The patients in this cohort were diagnosed between 1988 and 2015, before the MEST-C score was used in regular clinical practice [50]. Consequently, the lack of detailed data on biopsy findings, such as the MEST-C score, is another possible limitation in this study. However, there are also limitations to the use of the MEST-C score in clinical practice. A recent systematic review revealed that the reproducibility of all components in the MEST-C score is moderate to poor, especially in multi-institutional studies [51, 52]. Of all components, the T score (interstitial fibrosis/tubular atrophy) is most often tied to clinical outcomes, but even this component was proven inconsistently reliable as a prognostic indicator [51].

A great strength of this study is the long follow-up of up to 36 years. The cohort is large, consisting of 400 patients with biopsy-verified diagnosis and from multiple centers across Western Norway. We have gathered detailed information about kidney function, treatment and other data throughout the disease course, which has been made possible due to the long duration and detailed information provided in the Norwegian Kidney Biopsy Registry.

## Conclusions

In conclusion, our study sheds light on the complex and variable nature of IgAN progression, emphasizing the need for personalized treatment approaches and follow-up regimens, including proteinuria control. A deeper understanding of disease progression beyond initial diagnosis will be crucial in guiding therapeutic decisions and optimizing patient outcomes in the years to come.

## Supplementary Information

Supplementary Material 1.

## Data Availability

Owing to the sensitive nature of the data supporting the findings of this study, the data cannot be shared publicly for ethical reasons. The datasets used and/or analyzed in the current study are available from the corresponding author upon reasonable request.

## Abbreviations

IgAN: Immunoglobulin A nephropathy
KRT: Kidney replacement therapy
eGFR: Estimated glomerular filtration rate
KDIGO: Kidney Disease Improving Global Outcomes
IIgANPT: International IgAN Prediction Tool
ESKD: End-stage kidney disease
CKD-EPI: Chronic Kidney Disease Epidemiology Collaboration equation
CKD: Chronic kidney disease
IQR: Interquartile range
RAAS: Renin–Angiotensin–Aldosterone system
SGLT2: Sodium-glucose cotransporter 2
