# Supplementary material for: Lifetime progression of IgA nephropathy: a retrospective cohort study with extended long-term follow-up
Source: BMC Nephrol. 2025 Jan 21;26:32. doi: 10.1186/s12882-025-03958-y (PMC11749578; doi:10.1186/s12882-025-03958-y)
Supplement: Supplementary file 1 — Supplementary Material 1. [file 12882_2025_3958_MOESM1_ESM.docx]

Supplementary File

**Title**: Lifetime Progression of IgA Nephropathy: A Retrospective Cohort Study with Extended Long-term Follow-up

**Authors**: Mariell Rivedal^1^; Ole Petter Nordbø^1^, MSc; Yngvar Lunde Haaskjold^1,2^, MD; Rune Bjørneklett^1,3^, MD-PhD; Thomas Knoop^1,2^, MD-PhD; Øystein Eikrem^1,2^, MD-PhD

**Affiliations**:

^1^ Department of Clinical Medicine, University of Bergen, Bergen, Norway

^2^ Department of Medicine, Haukeland University Hospital, Bergen, Norway

^3^ Emergency Care Clinic, Haukeland University Hospital, Bergen, Norway

List of contents

[Figures 3](#_Toc187051588)

[**Supplementary Figure S1 – Patient selection** 3](#_Toc187051589)

[**Supplementary Figure S2 - The use of RAAS inhibitors in the cohort** 4](#_Toc187051590)

[Tables 5](#_Toc187051591)

[**Supplementary Table S1 - Comparison of eGFR slopes in our cohort with selected IgAN studies** 5](#_Toc187051592)

[**Supplementary Table S2 - Comparison of long-term outcomes between different countries** 7](#_Toc187051593)

[**Supplementary Table S3 - Therapeutic differences between patients with normal eGFR (≥ 90 mL/min/1.73 m^2^) at diagnosis** 8](#_Toc187051594)

[**Supplementary Table S4 - Therapeutic differences between patients with mildly decreased eGFR (60-89 mL/min/1.73 m^2^) at diagnosis** 9](#_Toc187051595)

[**Supplementary Table S5 - Therapeutic differences between patients with moderately/severely decreased eGFR (< 60 mL/min/1.73 m^2^) at diagnosis** 10](#_Toc187051596)

[Supplementary References 11](#_Toc187051597)

# **Figures**

## **Supplementary Figure S1 – Patient selection**


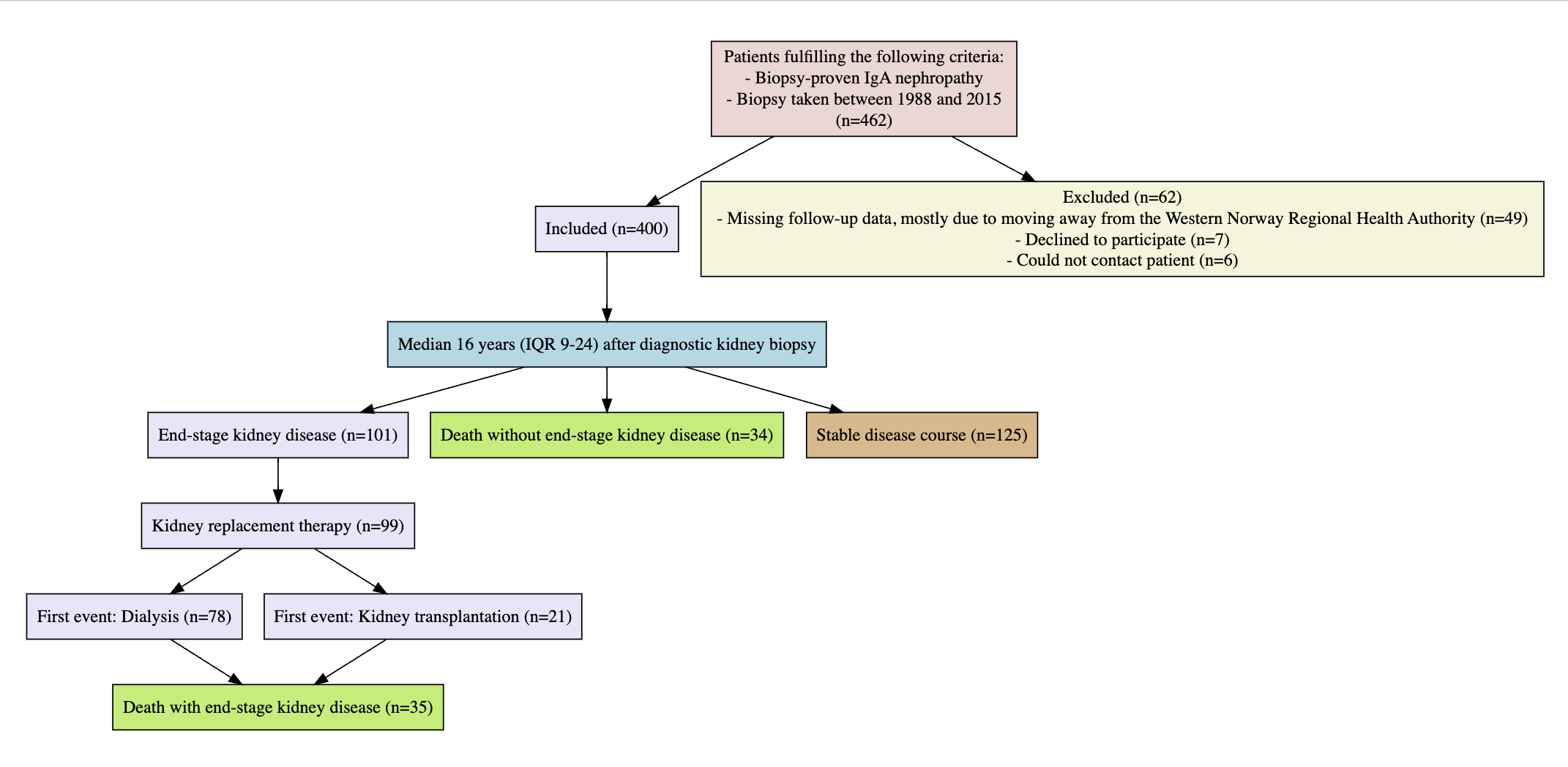


IQR = Interquartile range.

## **Supplementary Figure S2 - The use of RAAS inhibitors in the cohort**

**A**: Distribution of the proportion of the follow-up period in which RAAS inhibitors were given in each patient (n=184). **B**: Distribution of the most used types of RAAS inhibitors during each patient´s disease course. One square equals one patient. Candesartan (n=97) was the most used drug, followed by Losartan (n=41) and Enalapril (n=40). The least common drug was Telmisartan (n=1).

RAAS = Renin-angiotensin-aldosterone-system.

# **Tables**

## **Supplementary Table S1 - Comparison of eGFR slopes in our cohort with selected IgAN studies**

| **Cohort** | **Study design** | **eGFR slope**  **(mL/min/1.73 m^2^/year)** | **Follow-up**  **(years)** |
| --- | --- | --- | --- |
| Rivedal *et al*. – 2024 | Retrospective cohort study | -1.3 (-3.1, -0.4) | 16 (9, 24) |
| Pitcher *et al*. (1) – 2023 | Retrospective cohort study | −2.4 (−5.7, −0.6) | 5.9 (3.0, 10.5) |
| STOP-IgAN trial:  Rauen *et al*. (2)- 2015 (Intervention group) | Clinical trial  (Intervention: Corticosteroids) | 1.2 (-0.7, 3.1) | 7.4 |
| DAPA-CKD trial:  Wheeler et al. (3) – 2021 (Intervention group) | Clinical trial  (Intervention: Dapagliflozin) | −3.5 (0.5)• | 2.1 |
| DAPA-CKD trial:  Wheeler et al. (3) – 2021 (Placebo group) |  | −4.7 (0.5)• |  |
| TESTING trial:  Lv *et al*. (4) – 2022 (Intervention group) | Clinical trial  (Intervention: Corticosteroids) | 4.6 (1.2, 8.1) | 3.5 |
| ORIGIN trial:  Lafayette *et al*. (5) – 2024 (Intervention group) | Clinical trial  (Intervention: Atacicept) | 0.3 (-0.8, 1.5)* | 2.5 |
| Lafayette *et al*. (5) – 2024 (Placebo group) |  | -1.9 (-3.4, -0.4)* |  |

Values are median (interquartile range – if available), if not otherwise specified. •= Mean (SD). * = Mean absolute change (95% confidence interval). eGFR = Estimated glomerular filtration rate. Inspired by Lafayette *et al*. (6).

## **Supplementary Table S2 - Comparison of long-term outcomes between different countries**

|  | **Norway** | **Britain**  **(1)** | **Japan**  **(7)** | **China**  **(8)** |
| --- | --- | --- | --- | --- |
| Number of patients | 400 | 2439 | 1012 | 1155 |
| Gender (%) | Male: 72  Female: 28 | Male: 71  Female: 29 | Male: 41  Female: 59 | Male: 50  Female: 501 |
| Age at baseline (years) | Mean (SD):  38 (16) | Mean (SD): 41 (15) | Mean (SD):  33 (12) | Mean (SD):  31 (9) |
| eGFR at baseline (mL/min/1.73 m^2^) | Mean (SD):  74 (30) | Mean (SD): 55 (29) | Mean (SD):  79 (26) | Mean (SD):  89 (33) |
| Proteinuria at baseline (g/24 h) | Mean (SD):  1.8 (2.5)  Median (IQR): 1.0 (0.4-2.0) | Mean (SD): 2.4 (3.6)  Median (IQR): 1.5 (0.6-3.1) | Mean (SD):  1.2 (1.6) | Median (IQR): 0.9 (0.5-1.6) |
| Follow-up (years) | Mean (SD):  17 (9)  Median (IQR):  16 (9-24) | Mean (SD):  8 (7) | Mean (SD):  8 (7) | Median (IQR):  5 (4-7) |
| 10-year survival (%) | 79 | 54 | 84 | 83 |
| 20-year survival (%) | 67 | NE | 50 | 64 |

Values are presented as mean (SD), median (IQR), n or %. SD = Standard deviation. IQR = Interquartile range. NE = Not estimable.

## **Supplementary Table S3 - Therapeutic differences between patients with normal eGFR (≥ 90 mL/min/1.73 m^2^) at diagnosis**

|  | **Stable patient**  **(n = 60)** | **Unstable patient**  **(n = 78)** | ***p*-value** |
| --- | --- | --- | --- |
| At diagnosis | | | |
| RAAS inhibitor (yes) | 5 (8) | 23 (29) | 0.002 |
| Immunosuppression (yes) | 0 (0) | 1 (1) | 1.0 |
| During follow-up | | | |
| SGLT2 inhibitor (yes) | 0 (0) | 7 (9) | 0.02 |
| RAAS inhibitor (yes) | 32 (53) | 58 (75) | 0.01 |
| Immunosuppression (yes) | 5 (8) | 16 (21) | 0.06 |

Values are presented as frequencies (percentage). The *p*-value was based on Fisher’s exact test. RAAS = Renin-angiotensin-aldosterone-system. SGLT2 = Sodium glucose cotransporter 2.

## **Supplementary Table S4 *-* Therapeutic differences between patients with mildly decreased eGFR (60-89 mL/min/1.73 m^2^) at diagnosis**

|  | **Stable patient**  **(n = 37)** | **Unstable patient**  **(n = 92)** | ***p*-value** |
| --- | --- | --- | --- |
| At diagnosis | | | |
| RAAS inhibitor (yes) | 3 (8) | 17 (18) | 0.2 |
| Immunosuppression (yes) | 1 (3) | 0 (0) | 0.3 |
| During follow-up | | | |
| SGLT2 inhibitor (yes) | 3 (8) | 7 (8) | 1.0 |
| RAAS inhibitor (yes) | 25 (68) | 71 (78) | 0.3 |
| Immunosuppression (yes) | 2 (5) | 21 (23) | 0.02 |

Values are presented as frequencies (percentage). The *p*-value was based on Fisher’s exact test. RAAS = Renin-angiotensin-aldosterone-system. SGLT2 = Sodium glucose cotransporter 2.

## **Supplementary Table S5 - Therapeutic differences between patients with moderately/severely decreased eGFR (< 60 mL/min/1.73 m^2^) at diagnosis**

|  | **Stable patient**  **(n = 36)** | **Unstable patient**  **(n = 91)** | ***p*-value** |
| --- | --- | --- | --- |
| At diagnosis | | | |
| RAAS inhibitor (yes) | 4 (11) | 21 (23) | 0.1 |
| Immunosuppression (yes) | 0 (0) | 0 (0) | NE |
| During follow-up | | | |
| SGLT2 inhibitor (yes) | 2 (6) | 9 (10) | 0.7 |
| RAAS inhibitor (yes) | 24 (67) | 60 (69) | 0.8 |
| Immunosuppression (yes) | 6 (17) | 29 (33) | 0.08 |

Values are presented as frequencies (percentage). The *p*-value was based on Fisher’s exact test. RAAS = Renin-angiotensin-aldosterone-system. SGLT2 = Sodium glucose cotransporter 2. NE = Not estimable.

# **Supplementary References**

1. Pitcher D, Braddon F, Hendry B, Mercer A, Osmaston K, Saleem MA, et al. Long-Term Outcomes in IgA Nephropathy. Clin J Am Soc Nephrol. 2023;18(6):727-38.

2. Rauen T, Wied S, Fitzner C, Eitner F, Sommerer C, Zeier M, et al. After ten years of follow-up, no difference between supportive care plus immunosuppression and supportive care alone in IgA nephropathy. Kidney Int. 2020;98(4):1044-52.

3. Wheeler DC, Toto RD, Stefánsson BV, Jongs N, Chertow GM, Greene T, et al. A pre-specified analysis of the DAPA-CKD trial demonstrates the effects of dapagliflozin on major adverse kidney events in patients with IgA nephropathy. Kidney Int. 2021;100(1):215-24.

4. Lv J, Wong MG, Hladunewich MA, Jha V, Hooi LS, Monaghan H, et al. Effect of Oral Methylprednisolone on Decline in Kidney Function or Kidney Failure in Patients With IgA Nephropathy: The TESTING Randomized Clinical Trial. JAMA. 2022;327(19):1888-98.

5. Lafayette R, Barbour S, Israni R, Wei X, Eren N, Floege J, et al. A phase 2b, randomized, double-blind, placebo-controlled, clinical trial of atacicept for treatment of IgA nephropathy. Kidney Int. 2024;105(6):1306-15.

6. Lafayette RA, Reich HN, Stone AM, Barratt J. One-Year estimated GFR Slope Independently Predicts Clinical Benefit in Immunoglobulin A Nephropathy. Kidney Int Rep. 2022;7(12):2730-3.

7. Moriyama T, Tanaka K, Iwasaki C, Oshima Y, Ochi A, Kataoka H, et al. Prognosis in IgA Nephropathy: 30-Year Analysis of 1,012 Patients at a Single Center in Japan. PLOS ONE. 2014;9(3):e91756.

8. Le W, Liang S, Hu Y, Deng K, Bao H, Zeng C, et al. Long-term renal survival and related risk factors in patients with IgA nephropathy: results from a cohort of 1155 cases in a Chinese adult population. Nephrol Dial Transplant. 2011;27(4):1479-85.
